# The Allosteric Regulation of the DNA-Binding Domain of p53 by the Intrinsically Disordered C-Terminal Domain

**DOI:** 10.3390/ph19010124

**Published:** 2026-01-10

**Authors:** Shangbo Ning, Chengwei Zeng, Huiwen Wang, Junfeng Zhang, Yun Xue, Yunjie Zhao

**Affiliations:** 1 School of Medical Technology and Engineering, Henan University of Science and Technology, Luoyang 471023, China; jfzhang018@163.com (J.Z.); xueyun6688@163.com (Y.X.); 2 Institute of Biophysics and Department of Physics, Central China Normal University, Wuhan 430079, China; cwzengwuhan@mails.ccnu.edu.cn; 3 School of Physics and Engineering, Henan University of Science and Technology, Luoyang 471023, China; huiwenwang@haust.edu.cn

**Keywords:** intrinsically disordered proteins, allosteric mechanism, free energy landscapes, molecular dynamics, MM-PBSA

## Abstract

**Background**: Intrinsically disordered regions (IDRs) within proteins often act as pivotal linkage units for the interaction of functional domains. The p53 tumor suppressor protein contains intrinsically disordered N-terminal and C-terminal domains (NTD and CTD), playing crucial regulatory roles in cellular processes. Furthermore, experimental approaches have encountered challenges in elucidating the structural regulation by the IDRs. **Methods**: In this work, we employed microsecond-scale molecular dynamics simulations to explore the allosteric regulation mechanism of the p53 DNA binding domain (DBD) induced by the CTD and the DNA binding. Subsequently, we integrated dynamic cross-correlation analysis with binding free energy calculations to evaluate the interaction between the CTD and DNA. **Results**: The free energy landscapes (FELs) were utilized to identify the conformational ensemble of the p53 DBD. The FELs revealed that the CTD enhances the allosteric regulatory mechanisms. **Conclusions**: Firstly, the conformation of DBD changes on the S6-S7 loop and L1 upon DNA binding. Then the CTD directly interacts with DNA and further regulates the allosteric network (involving the S6-S7 loop, L1 loop, S4, S10, H1, and H3) to promote the binding of DBD to DNA. The allosteric mechanisms presented in this work will provide new insights into the functional mechanisms of the p53 CTD and inform the rational design of p53-targeted drugs.

## 1. Introduction

Intrinsically disordered regions (IDRs) in proteins are segments of at least 30 amino acids in length that lack stable three-dimensional structures under physiological conditions [1,2]. IDRs play crucial roles in various biological functions, including molecular recognition, protein–protein interactions, and regulation of cellular processes [3,4,5]. Based on previous studies, approximately 44% of human proteins contain disordered regions [6]. These regions are characterized by their dynamic nature, which allows them to sample a wide range of conformations and interact with multiple partners, often through short linear motifs [7,8]. IDRs are enriched in polar and charged amino acids, such as serine, threonine, and glutamic acid [9,10]. These characteristics contribute to the disordered state due to reduced hydrophobic interactions and a higher net charge.

**Figure 1:**
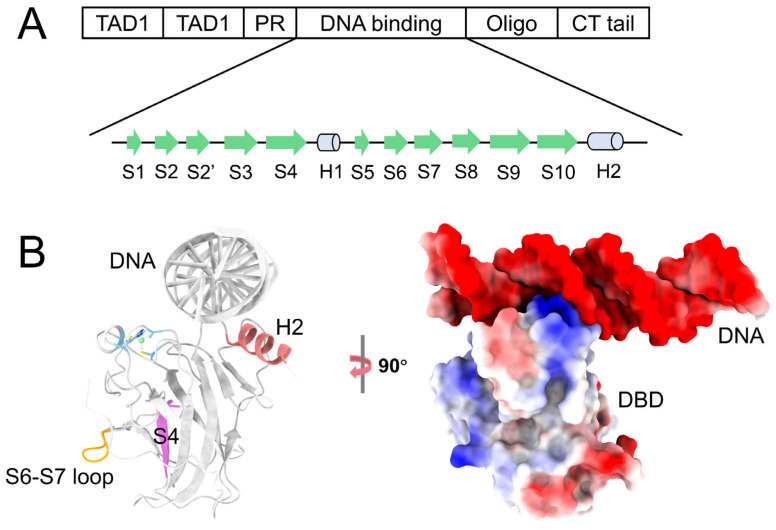
Structural domains of p53 and the conformation of its DNA-binding domain (DBD). (**A**) Schematic representation of the domain architecture of p53. The N-terminal domain (NTD) comprises two transactivation domains (TAD1: residues 1–40; TAD2: 40–60) and a proline-rich domain (PRD: 60–90). The central core corresponds to the DNA-binding domain (DBD: 94–312). The C-terminal domain (CTD) includes an oligomerization domain (OD: 323–355) and a basic, lysine-rich regulatory tail (364–393). (**B**) Structure of the p53 DBD in complex with double-stranded DNA. The DBD adopts a β-sandwich fold, with the DNA-binding interface primarily formed by helix H2. This region contains negatively charged residues critical for DNA interaction.

The p53 tumor suppressor protein is a transcription factor that maintains cellular stability and prevents cancer [11]. The p53 modulates fundamental cellular processes through protein–protein interactions [12]. Structurally, the p53 comprises four subunits of 393 amino acids and contains IDRs in both N-terminal and C-terminal domains. As shown in Figure 1, the N-terminal domain (NTD, residues: 1–90) includes two transactivation domains (TAD1, residues: 1–40, TAD2, residues: 40–60) and a proline-rich region (PRR, residues: 60–90) [13]. TAD1 contains the primary binding sites for MDM2 and MDMX. TAD2 is involved in transcriptional activation and protein interactions, such as with the RPA protein [14]. Mutations in TAD1 and TAD2 are required to eliminate tumor suppressor function [15]. TAD1 and TAD2 also contain multiple phosphorylation sites that regulate the degradation and activity of p53 in response to stress [16]. The DNA binding domain (DBD: 94–312) is essential for sequence-specific DNA binding and is the target of most point mutations in cancer [17]. The C-terminal domain (CTD, residues: 312–393) contains an oligomerization domain (residues: 323–355) and a lysine-rich C-terminal tail (residues: 364–393), which includes sites for acetylation, methylation, and phosphorylation [18].

Functionally, the DBD is the main functional region of p53 that is particularly crucial for the cytoplasmic and nuclear functions of the protein [19]. In addition, the intrinsically disordered N-terminal and C-terminal domains also have important regulatory functions. For instance, the N-terminal transactivation domain of p53 may be involved in multiple functions. TAD1 and TAD2 interact with transcription factors such as TBP, TFIID, and coactivators like p300/CBP, regulating signaling pathways during DNA damage [20]. The NTD is phosphorylated at multiple sites, exerting diverse functions through the involvement of various protein kinases [21]. The CTD may modulate DNA binding, and the resulting stimulation plays a key role in subsequent signaling pathways [22,23]. The acetylation of the CTD by p300/CBP can stimulate specific DNA binding in vitro. The CT tail interacts with the DBD in subunit-subunit interactions, promoting p53 oligomerization [24]. The CTD can bind to non-specific DNA and enable p53 to slide along the DNA, which is an important step in finding specific binding sites [25]. Therefore, a series of molecular experiments implies the key roles of these IDRs in DNA binding and regulation.

Previous studies for the p53 structure primarily focus on the DBD and DNA binding [26,27,28]. Lambruschini et al.’s study adopted classical molecular dynamics (MD) simulations and Metadynamics to unveil the allosteric mechanisms of the p53 DBD during DNA binding [29]. The local perturbation within the p53 structural network can lead to the transmission of a protein structure. On the other hand, a recent study suggests that the p53 NTD can inhibit binding to DNA and increase p53 DNA binding specificity [13]. The observations for CTD lead to a model for allosteric regulation of p53 and modulate the conversion between latent and activated forms of p53 [21]. However, the mechanism by which the CTD regulates the structure and function of the DBD remains unclear. To understand the regulatory mechanism of p53 functional activity, obtaining the structural details and dynamic characteristics between CTD and DBD upon DNA binding is urgent.

In this work, the conformational dynamics of the p53 DBD and the DNA binding affinity upon CTD-DNA binding were studied by all-atom molecular dynamics (MD) simulations. We observe a significant correlation between the structural characteristics of the S6-S7 loop and the global allosteric network of p53. These conformational shifts are pivotal to the functionality of p53, potentially impacting both its DNA binding affinity and its capacity for transcriptional regulation. Furthermore, the CTD engages in direct interactions with DNA, thereby amplifying the DNA-binding affinity. The CTD further promotes allosteric regulation and conformational transition by stabilizing DBD binding to DNA. The global allosteric network of DBD comprises residues F212, R280, Q167, V122, R156, and R267. Our results indicate that the CTD regulates the global allosteric network of the DBD, including the S6-S7 loop, etc. The structural details provided in this article highly coincide with the experimental observations. These discoveries provide novel insights into the allosteric regulatory mechanisms of p53 and the disease pathology.

## 2. Results

The structural ensemble of p53 DBD and dynamic changes is crucial for understanding the mechanisms of transcriptional regulation and other functions. Previous studies suggested that the conformation of the DBD changes after binding to DNA [29]. p53_DBD as the core DNA-binding domain alone, and full-length p53 as the DBD flanked by both the N-terminal and C-terminal intrinsically disordered regions (IDRs). Therefore, these IDRs, particularly the CTD, may not merely be passive tethers but actively modulate the structure, dynamics, and allosteric regulation of the DBD upon DNA binding. To elucidate the regulatory role of the CTD region for the p53 and DNA binding, we integrated microsecond-scale unbiased dynamics, dynamic correlation networks, and free-energy landscapes to analyze the conformational dynamics and thermodynamic changes.

**Figure 2:**
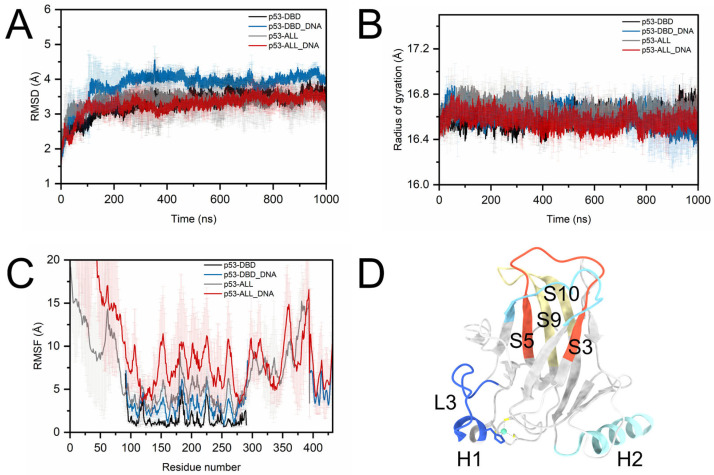
Conformational dynamics of p53_DBD_ and p53_ALL_ upon DNA binding. (**A**) Root-mean-square deviation (RMSD) of the DNA-binding domain (DBD) in the four simulated systems: p53_DBD_, p53_ALL_, p53_DBD-DNA_, and p53_ALL-DNA_. (**B**) Radius of gyration (Rg) of the DBD across the four systems. (**C**) Root-mean-square fluctuation (RMSF) of backbone heavy atoms in the four systems. (**D**) Structural mapping of regions exhibiting significant conformational flexibility upon DNA binding, with residues color-coded according to their RMSF values.

To observe the structural property of the core domain upon DNA binding, we characterized the dynamic changes in DBD in both p53_DBD-DNA_ and p53_ALL-DNA_ models. As shown in Figure 2A, we calculated the root-mean-square deviations (RMSDs) of the p53_DBD_ and p53_ALL_ structures upon DNA binding. After a 500 ns equilibration, the RMSD values for all four systems fluctuated within a certain range, suggesting that the system had generally reached a stable state. Notably, the RMSDs of the DBD region in both experimental and theoretical models show significant changes upon DNA binding. The DNA binding induces larger fluctuations in the overall structure of DBD. The R_g_ value decreases from 16.6 Å to 16.4 Å (p53_DBD-DNA_) and the p53_ALL-DNA_ decreases from 16.8 Å to 16.6 Å upon DNA binding (Figure 2B). It suggests the protein structures are more compact upon binding to DNA, aligning with a transition to a more stable conformation. In our analysis of the root-mean-square fluctuations (RMSFs) of amino acid residues within the p53 protein, significant fluctuations are observed in the heavy atoms of residues V143-M160, H179-H193, V218-T230, T253-N268, and A276-K291 (Figure 2C,D). These fluctuations indicate that the DBD adopts different conformations upon DNA binding.

### 2.1. The Internal Contact Within the p53 DBD Changes upon DNA Binding

**Figure 3:**
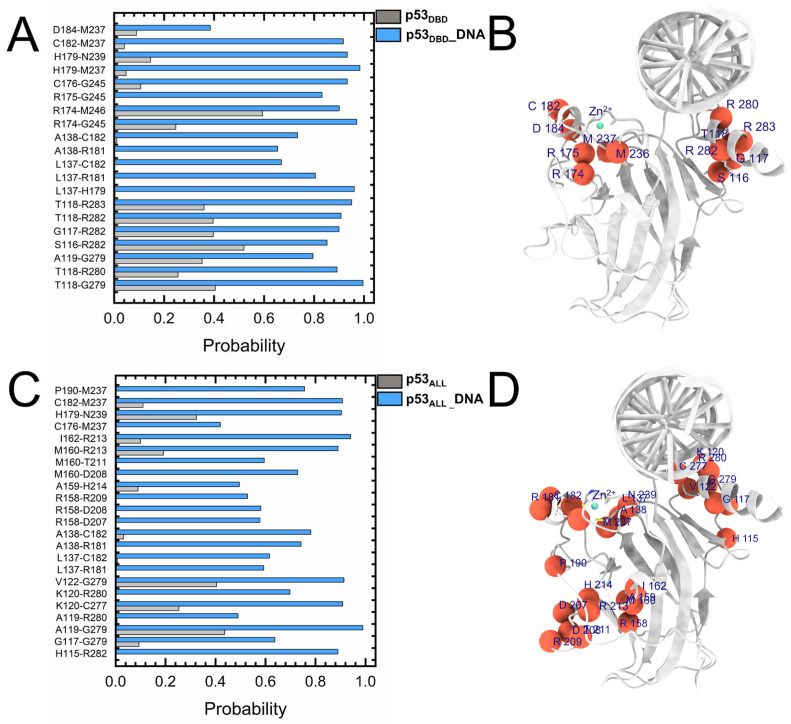
Changes in internal residue contacts within the p53 DBD upon DNA binding. (**A**) Residue pairs exhibiting high contact probabilities in the p53_DBD-DNA_. (**B**) Residues contributing to altered internal interactions within the DBD in the p53_DBD-DNA_ system. (**C**) Residue pairs with high contact probabilities in the p53_ALL-DNA_. (**D**) Residues associated with changes in DBD internal interactions in the p53_ALL-DNA_ system.

The residue contact maps are employed to delineate the interaction changes within the p53 DBD upon DNA binding (Figure 3, Figures S2 and S3). As shown in Figure 3A, the contact probability of the DBD (p53_DBD-DNA_) increases by 60.0–80.0%. The dynamic regions are mapped onto the three-dimensional structure of the DBD (Figure 3B), mainly located at the zinc finger binding region and the DNA binding interface (A276-K291). In the contact map of the p53_ALL-DNA_, the contact probability of the DBD increases by over 60.0% upon DNA binding compared to the p53_DBD-DNA_ simulations (Figure 3C,D). Consequently, the interaction network within the DBD also undergoes significant changes upon DNA binding, with a notable increase in internal contacts. The internal correlations of the DBD are further enhanced in the p53_ALL-DNA_ simulation.

To further elucidate the binding mode of the DBD with DNA and the conformational changes in the DBD upon DNA binding, the free energy landscapes were employed to analyze the structural ensemble of the DBD. The x-axis represents the RMSDs of the p53 DBD, and the y-axis represents the R_g_ of the DBD (Figure S4), describing the structural fluctuation and stability. Three major minima of the DBD conformation (states 1, 2, and 3) are characterized by the free energy landscapes upon DNA binding, and the representative structures of the minima are given in Figure S4. The population of structures in state 1 is 57.5% (Figure S4A). The population of state 2 is 68.2% upon DNA binding (Figure S4B). Moreover, a new sub-conformation (state 3) appears after DNA binding, and the population is about 19.2%. The FEL of the p53_DBD-DNA_ suggests that the DNA binding promotes the population of structures 2 and 3 (the closed state of the S6-S7 loop), and the S6-S7 loop is closer to the flanking S4 β-sheet. Previous studies have also pointed out similar observations regarding the structural heterogeneity of the S6-S7 loop [26,29]. In addition, the conformations of the L1 loop in states 2 and 3 (Figure S4C) are closer to the DNA binding site (H2 helix), indicating that L1 also plays a role in DNA binding. We further selected the distances between two sets of amino acid residues as collective variables to probe the correlation between the conformational changes in the S6-S7 loop and allosteric regulation. As shown in Figure S5A,B, the distance between the two groups of amino acid residues is approximately 13.0 Å. The FEL shows subtle changes in the two sets of distances. Therefore, the DNA binding alters the free energy landscape of the p53 DBD and promotes population shifts in the conformation (S6-S7 loop).

### 2.2. The CTD Stabilizes the Specific Interactions Between p53 and DNA

**Figure 4:**
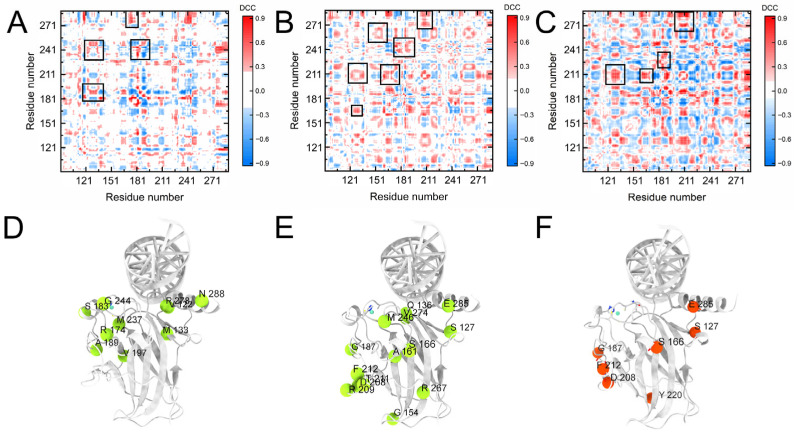
Dynamic correlation changes within the DBD upon DNA binding. (**A**–**C**) Difference maps of residue–residue dynamic correlation coefficients. Blue and red grids indicate decreased and increased correlations, respectively. The black boxes highlight regions of significantly enhanced positive correlation within the two systems. (**A**) p53_DBD-DNA_ vs. p53_DBD_. (**B**) p53_ALL-DNA_ vs. p53_ALL_. (**C**) p53_ALL-DNA_ vs. p53_DBD-DNA_. (**D**–**F**) Structural mapping of residues involved in dynamic network reorganization. (**D**) p53_DBD-DNA_ vs. p53_DBD_. (**E**) p53_ALL-DNA_ vs. p53_ALL_. (**F**) p53_ALL-DNA_ vs. p53_DBD-DNA_. Regions with altered dynamic correlations in p53_DBD-DNA_ are localized near the DNA-binding interface, whereas changes in p53_ALL-DNA_ involve the entire DBD.

To analyze the regulation of CTD for the internal network of the DBD, we calculated the dynamic cross-correlation coefficients (DCCs) of DBD and the difference in the DCCs between the p53_DBD-DNA_ and p53_ALL-DNA_ systems upon DNA binding (Figures S6 and Figure 4). Positive values (red) indicate an increase in the magnitude of correlation, while negative values (blue) indicate a decrease. In the DCCs of the p53_DBD-DNA_ system, the correlations of multiple regions increase by more than 50.0% upon DNA binding (Figure 4A,B and Figure S7). The position of these regions is consistent with the residue contact map. Compared to the residue contact map, the DCCs are more acute for the motion of the internal networks. For the p53_ALL-DNA_, the network of the DCCs within DBD is amplified upon DNA binding and involves the global DBD (Figure 4D,E and Figure S8). Meanwhile, Figure S2 shows the interactions between the CTD and DNA, suggesting that the CTD may regulate the allosteric networks by binding to DNA. We also compared changes in the DCC network of DBD following the incorporation of the CTD upon DNA binding. As shown in Figure 4C,F, the incorporation of CTD resulted in significantly enhanced positive correlations among four regions within DBD (including S127, S166, G187, F212, E285, etc.). This result likely corresponds to a more active conformational state that facilitates DNA binding.

**Figure 5:**
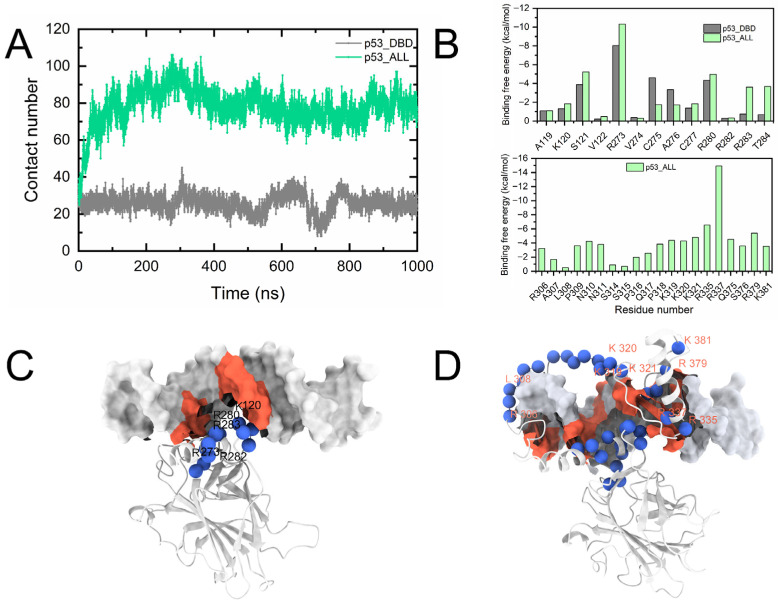
Molecular interactions between p53 and DNA. (**A**) Coordination number of residue–DNA contacts in the p53_DBD-DNA_ and p53_ALL-DNA_ systems. (**B**) Per-residue decomposition of binding free energy for the p53_DBD-DNA_ and p53_ALL-DNA_ interactions. (**C**) Structural view of the interaction interface between the DBD and DNA. (**D**) Structural view of the interaction interface between the C-terminal domain (CTD) and DNA.

As shown in Figure S2B, the contact probability of the DNA binding interface (H2 helix) with DNA is only around 0.5. In contrast, the contact probability of amino acid residues near the H2 helix with DNA in the p53_ALL-DNA_ system has increased to 0.8–0.9 (corresponding to the black grid). To further examine the impact of the CTD on the stability of p53 binding to DNA, we compared the residue contact coordination numbers between proteins and DNA in both the p53_DBD-DNA_ and p53_ALL-DNA_ systems. In Figure 5A, the result shows that the coordination number in the p53_ALL-DNA_ system increases from around 25 (p53_DBD-DNA_) to 80 (p53_ALL-DNA_). In the full-length p53-DNA system, the interaction between the DBD and DNA is enhanced, and the CTD provides a significant contribution to the binding free energy with DNA (Figure 5B). These results suggest that the CTD enhances the binding interaction between p53 and DNA (Figure 5C,D) and also confirm the role of the CTD in regulating DNA binding, as mentioned in experiments [22].

**Figure 6:**
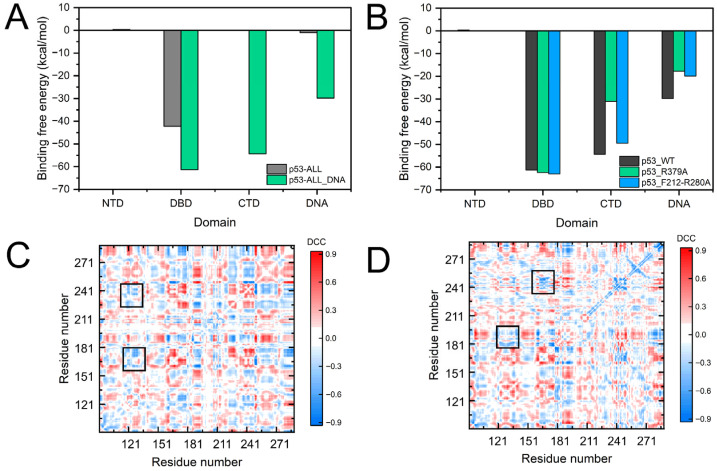
Binding free energy and DCC analysis of p53–DNA interactions in different structural contexts and mutants. (**A**) Domain-wise contributions to the total binding free energy between p53 and DNA. Values are shown for the full-length protein (p53_ALL_) and its DNA-bound state (p53_ALL-DNA_). Negative values indicate favorable binding contributions. (**B**) The plots compare wild-type (p53_WT) with two mutants: p53_R379A and p53_F212–R280A. (**C**,**D**) Difference maps of residue–residue dynamic correlation coefficients. (**C**) p53_ALL-DNA_ (R379A) vs. p53_ALL-DNA_. (**D**) p53_ALL-DNA_ (F212A/R280A) vs. p53_ALL-DNA_.

We also calculated the binding free energy of both systems and performed an energy decomposition of the contributions from amino acid residues during the binding. The binding free energy represents the binding affinity between two molecules. The negative value of the binding free energy for receptors and ligands in the gas or aqueous phase indicates a favorable binding, while a positive value suggests an unfavorable interaction. We first calculated the binding free energy between the p53_DBD_ and DNA. As shown in Table S1, the binding free energy between p53_DBD_ and DNA is −41.3 kcal mol^−1^. The main contribution of the binding energy between p53_DBD_ and DNA is derived from the electrostatic interaction energy, which is favorable in the gas phase. In the case of the p53_ALL-DNA_, the result shows that the binding free energy significantly increases to −161.1 kcal mol^−1^ (Table S2). To study the binding mechanism between the CTD and DNA, we performed per-residue energy decomposition of the binding free energy between the p53_ALL_ (p53_DBD_) and DNA. Figure 5B shows the significantly enhanced binding free energy contributions in the p53_ALL-DNA_ compared to the p53_DBD-DNA_. The result shows that the enhanced energy contributions are mainly derived from the CTD, primarily located in residues R306-K321, R335, and R379 of the CTD (Figure 5D). The simulation of p53_ALL-DNA_ shows a more favorable contribution (p53 and DNA) to the p53_DBD-DNA_. These results support the experimental proposition that the p53 CTD plays a substantial role in DNA binding [24]. As shown in Figure 6A, the CTD contributes a more significant favorable energy (−54.32 kcal/mol), specific residues in the NTD provide an insignificant contribution (0.35 kcal/mol), consistent with the increased correlations observed in Figure 5. The p53 DBD specifically binds to DNA through the H2 helix and L1 loop, while the CTD further promotes the interaction between p53 and DNA.

### 2.3. The CTD Induces Allosteric Regulation of DBD by Promoting DNA Binding

**Figure 7:**
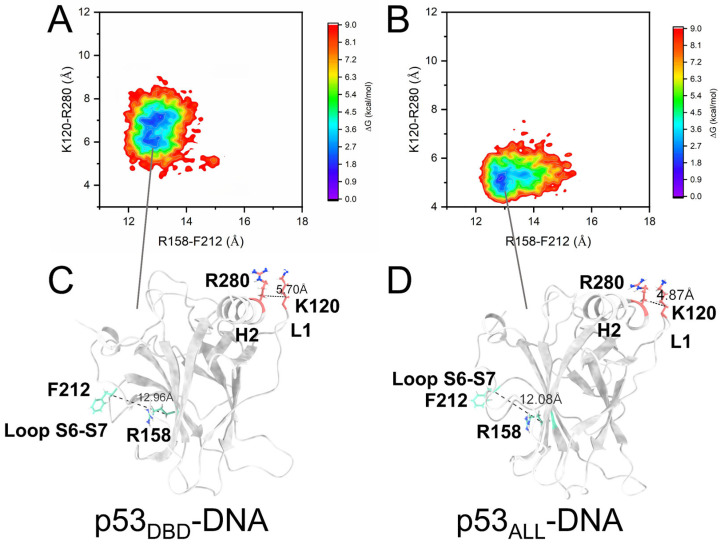
Conformational free-energy landscapes of the S6-S7 and L1 loops in p53 upon DNA binding. (**A**) Two-dimensional free-energy surface for p53_DBD-DNA_, projected onto the Cα–Cα distances of R158–F212 (x-axis) and K120–R280 (y-axis). This landscape reflects conformational sampling of the S6-S7 and L1 loops. (**B**) Corresponding free-energy landscape for p53_ALL-DNA_, described by the same distance variables. (**C**,**D**) Representative conformational motions of the S6-S7 and L1 loops extracted from the energy minima in (**C**) p53_DBD-DNA_ and (**D**) p53_ALL-DNA_.

To understand the regulation mechanism of the conformational changes in DBD and DNA binding induced by the CTD, we also constructed the free energy landscape for the p53_ALL_/p53_ALL-DNA_. In Figure S9, the RMSD of the DBD increased to 3.6 Å, and the R_g_ decreased to 16.5 Å upon DNA binding. Simulations by Pradhan et al. suggested that the conformational state of the L1 loop may be related to the motion within the S6-S7 loop [26]. In our simulations, both the p53_DBD_ and p53_ALL_ simulations show the conformational change in the L1 loop upon DNA binding. We used two CVs to concurrently describe the conformational dynamics of the S6-S7 loop and L1 loop. The x-axis represents the distance between the CA atoms of residues R158 and F212, and the y-axis represents the distance between the CA atoms of K120 and R280. In the simulation of p53_DBD-DNA_ (Figure 7A), the distance between the CA atoms of R280 and K120 is 5.70 Å after DNA binds to DBD, and the distance between the CA atoms of F212 and R158 is 12.96 Å. In the p53_ALL-DNA_ simulation (Figure 7B), the distance between the CA atoms of R280 and K120 further decreases to 4.87 Å after CTD binds to DNA, indicating that the L1 and H2 move closer together. This conformation facilitates the simultaneous insertion of L1 and H2 into the DNA major groove. Meanwhile, the distance between F212 and R156 also shortens to 12.08 Å (Figure 7C,D), and the conformational change in the S6-S7 loop promotes allosteric regulation within the DBD, which is consistent with previous simulation studies of the DBD region [29]. Interestingly, both K120 and R280 are located at the DNA-binding interface and carry positive charges. The decreased distance between these residues enhances DBD-DNA binding affinity through favorable electrostatic interactions and promotes the second transition of the DBD structure.

The conformational states of the S6-S7 loop and L1 loop upon DNA binding induce the more compact structure of the p53 DBD, which is favorable for the internal network connections. The DCC analysis (Figure 4C) shows the correlation nodes within the DBD of the p53_ALL-DNA_. The structural network of the DBD is mainly composed of F212 from the S6-S7 loop, E285 from the H2 helix, S166 from H1, and S127 from the L1 loop (Figure 4F). Therefore, the amino acids of S4 and S10 act as connecting units between the S6-S7 loop, H2 helix, and the L1 loop. These results indicate that the conformation of DBD binding to DNA is mediated by the conformational changes in the global network. The conformation shift (S6-S7 loop) is crucial for the recruitment of biological partners. The CTD further promotes the allosteric regulation of the DBD conformation and the subsequent protein–protein interactions. Moreover, the CTD-mediated stabilization of the DBD-DNA complex and the modulation of its conformational dynamics likely facilitate the subsequent recruitment of coactivators (such as MDM2/p300) and other transcription machinery, which are essential for p53′s transcriptional activity [21,24].

### 2.4. In Silico Mutagenesis Validates the Allosteric Network

To validate the impact of the allosteric network on the interaction between p53 and DNA binding, we calculated the DCC maps and binding free energies for these mutants. The results show a significant reduction in both long-range correlations of DBD (Figure 6C,D) and the favorable binding energy contribution from the mutated regions (Tables S3 and S4). The binding free energy contributions from the domains reveal that the R379A mutation significantly weakens the direct binding free energy contribution of the CTD to DNA (Figure 6B). In the R379A mutation simulation, the binding free energy contribution of the CTD region to DNA decreased from −54.32 kcal/mol to −31.00 kcal/mol. Similarly, in the F212A/R280A double-site mutation simulation, the binding free energy contribution of CTD also decreased to −49.50 kcal/mol. Furthermore, compared to the wild type, the DBD of both mutants, particularly R379A, shows significantly negative correlations. These results suggested the regulatory role of the CTD basic region and the DBD allosteric network in DNA binding. These results suggested the regulatory role of the CTD basic region and the DBD allosteric network in DNA binding.

We also evaluated the structural changes induced in the intrinsically disordered N- and C-terminal domains upon DNA binding. As shown in Figure S10A,B, DNA binding induces a measurable increase in helical content within both terminal domains. Specifically, in the CTD (residues 340–360), the average helicity increases in the p53_ALL-DNA_ complex compared to the DNA-free state (p53_ALL_). The NTD also shows localized stabilization, particularly in the region spanning residues 12–23. This indicates a shift towards a more ordered conformation in both IDRs upon interaction with DNA. Our site-directed mutagenesis simulations provide clear evidence that destabilizing the allosteric network impairs this ordering. As visualized in Figure S10D, the R379A mutation, targeting a critical CTD anchor point, causes a decrease in helicity (residues 365–370) and increases conformational fluctuations in the NTD (Figure S10C). The F212A/R280A double mutation within the core DBD disrupts the long-range coupling, leading to a substantial reduction in helical stability.

## 3. Discussion

The IDRs within the protein play a crucial regulatory role in key biological functions by acting as linker units in the binding process of protein complexes, contributing to structural flexibility. The p53 protein contains highly disordered N-terminal and C-terminal sequences, and the DNA-binding domain. The confirmation of DBD is critical for binding biological partners and subsequent transcriptional regulation. Previous studies mainly focus on the conformational and functional changes in the DBD upon DNA binding. The experiment revealed the different properties of p53 NTD and CTD in DNA binding [21,22,23]. However, there is still a lack of structural details about the regulation of the internal network within DBD and DNA binding.

**Figure 8:**
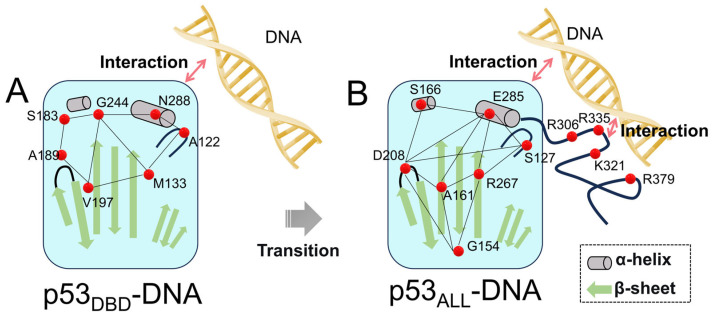
Proposed regulatory model of DNA and CTD binding on p53 DBD stability. (**A**) When DNA binds only to the DBD (CTD unbound), a transient interaction network forms within the DBD, resulting in partial complex stabilization. (**B**) Cooperative binding of DNA to both the DBD and CTD induces extensive strengthening of the DBD internal interaction network, leading to full stabilization of the p53–DNA complex.

In this paper, we investigated the regulation of the internal network of the DBD by the CTD upon DNA binding. We employed microsecond-scale MD simulations to establish the correlation between the DBD conformation and the p53 CTD. Some of our results are consistent with the previous enhanced sampling simulations. In addition, the simulations of p53_ALL-DNA_ elucidate the regulatory roles of the CTD upon DNA binding. Upon DNA binding to the DBD (Figure 8), the internal correlation of the DBD begins to increase, revealing a preliminary allosteric network. Subsequently, as the CTD region interacts with DNA, the binding between the DBD and DNA is further stabilized, accompanied by an expansion of the regions involved in the internal allosteric network. Therefore, DNA binding serves as the first critical step in the allosteric regulation within p53, while CTD-DNA interaction constitutes the second step in modulating the internal allosteric network. The DBD adopts a more compact conformation upon DNA binding, the S6–S7 loop transits to a closed state, and the L1 loop shifts closer to the H2 helix to facilitate DNA binding. The CTD directly interacts with DNA through electrostatic interactions, expanding the interface of p53 binding with DNA. The CTD modulates the DNA-binding affinity and the structural conformation through global allosteric regulation. Thus, the CTD-induced stabilization of the DBD conformation and the identified allosteric network likely pre-organize or create interfaces that facilitate the recruitment of transcriptional co-activators (like MDM2/p300) [30].

Additionally, we discussed the robustness of the predicted p53 structure in the regulatory mechanism of the CTD. We employed Rosetta and Swiss-Model to predict the full-length protein structure of p53 and performed 500 ns MD simulations for each. As shown in Figure S11A, the positions of the CTD region in the initial structural models of p53 predicted by different methods are different. However, the CTD region ultimately binds to DNA in all cases during dynamics simulations (Figure S11B). This is due to the abundance of basic amino acids in the CTD region, and the electrostatic interactions facilitate its binding near DNA. Moreover, this interaction becomes further stabilized as the dynamics trajectory progresses (Figure S11C,D).

The methodology of this work also has some limitations in calculating binding free energy. MM-GBSA provides relative trends rather than absolute binding affinities. The large energy contribution of the CTD should be interpreted qualitatively, highlighting its significant stabilizing role, not as a precise thermodynamic measurement, particularly in handling solvent and ion effects for charged systems like DNA-protein complexes. Therefore, future therapeutic strategies could potentially regulate the transition of the p53 protein between active and inactive states through rational drug design. By inhibiting the allosteric network and DNA-binding activity of p53, it may be possible to suppress disease progression.

## 4. Materials and Methods

**Figure 9:**
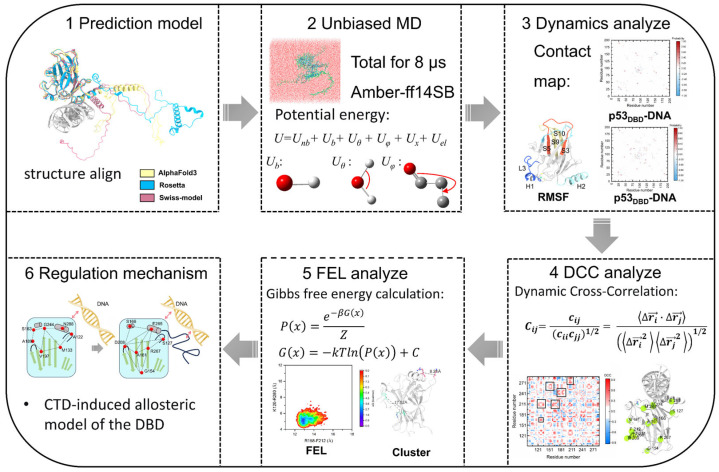
Workflow for unbiased molecular dynamics simulations and post-analysis of conformational dynamics and binding energetics.

We integrated dynamics and thermodynamic analyses to explore the allosteric mechanism of p53 CTD regulating DBD structure and DNA binding. The workflow of the unbiased molecular dynamics (MD) method is illustrated in Figure 9. First, we employed multiple structure prediction software to perform structural modeling of the p53 protein, facilitating subsequent assessment of the robustness of the structural mechanisms. Second, unbiased MD simulations were performed for the four p53 complex systems. Third, residue contact maps and atomic fluctuations were analyzed to characterize the binding interactions between the p53 DBD/CTD and DNA. Fourth, dynamic cross-correlation network analysis was applied to examine changes in the internal interaction network of DBD upon DNA and CTD binding. Fifth, the free energy landscape (FEL) analysis was performed to identify key conformations associated with CTD-mediated regulation of the DBD network, followed by cluster analysis. Finally, the allosteric regulatory mechanism of DBD induced by CTD binding was summarized.

### 4.1. Simulation Protocols

The structure of the DBD (p53_DBD_: 92–290) was obtained from the experimental structure (PDB ID: 2XWR) [31]. The complex structure of the DBD binding to DNA (p53_DBD-DNA_: 92–290, in complex with DNA) was derived from the X-ray crystal structure (PDB ID: 3KMD) [32]. We utilized AlphaFold3 to predict and construct the full-length p53 protein structure (Figure S1) [33]. Subsequently, we employed Chimera-1.15 to build the zinc finger for the p53 protein (p53_ALL_: 1–393) [34]. Finally, we referred to the experimental structure (PDB ID: 3KMD) and used Chimera-1.15 to construct the complex structure of the p53_ALL_ protein bound to DNA (p53_ALL-DNA_). At this point, we have constructed four molecular dynamics simulation systems (Table 1) to investigate the conformational changes in the DBD upon DNA binding and the effect of the CTD on the binding interaction between the DBD and DNA. The systems were solvated in a dodecahedral box using the TIP3P water model and neutralized with Na^+^ and Cl^−^ ions to balance the charges within the system [35]. The periodic boundary conditions with a minimum distance were set as 12.5 Å between protein and box edges. We used the AMBER/ff14SB force field, including parameters for proteins and DNA [36]. The Zinc AMBER force field (ZAFF) was adopted for zinc fingers [37].

We performed two independent, unbiased molecular dynamics (MD) simulations for each system, with each simulation generating 1 μs of trajectory, resulting in a total of 8 μs of MD simulation data using identical parameters [38]. The key parameter settings are detailed below. The systems were first minimized by using the steepest descent method until the maximum force (1000.0 kJ/mol/nm) or the minimization step reached 20,000 steps. Then, the system was heated to 300 K in two steps with the Nośe-Hoover thermostat method at 100 ps in the NVT ensemble [39]. No restraints were applied during the energy minimization and heating phases of the MD simulations. Next, another 100 ps MD equilibration in the NPT ensemble was performed at the pressure of 1 atm, which was coupled by the Parrinello–Rahman barostat method [40]. The integrating step was 2 fs in all the simulations. The Particle Mesh Ewald method (PME) was used to calculate the long-range electrostatic interactions with a van der Waals cut-off of 10 Å [41]. The hydrogen bonds are constrained by the SHAKE algorithm [42].

To validate the allosteric network in p53, we established additional simulations for two key mutant systems suggested by the network analysis: one with a mutation in the CTD (R379A) and another with a double mutation affecting a DBD interface residue (F212A/R280A).

### 4.2. Free Energy Analyses

#### 4.2.1. Construction of the Free Energy Landscape

The free energy landscapes (FELs) represent the potential of the mean force of the systems. We strategically employed two distinct sets of collective variables (CVs) to evaluate the dynamic fluctuations within the p53 DBD. The initial set encompasses the root mean square deviation (RMSD) and the radius of gyration (R_g_) of the p53 DBD. These CVs can reflect the correlation between the conformational changes in p53 DBD and the stability of the internal network. The CVs are defined by the distances between the CA atoms of residues R158-F212, R156-D208, and K120-R280 within the p53 DBD. The distances between the CA atoms of amino acid residues on the S6-S7 loop and the L1 loop describe the conformational changes in the DBD allosteric network under the regulation of CTD. The calculation of free energy employed the GROMACS tool, which adeptly integrates histogram analysis with Boltzmann statistics to elucidate the free energy landscape of molecular systems [43]. The FELs are derived from the probabilities of the system being in specific states, which are calculated from the histogram data. The relationship is given by the Boltzmann distribution:

(1) $$P\left(x\right)=\frac{e^{-\beta G\left(x\right)}}{Z}\;$$

where *P*(*x*) is the probability of the system being in state *x*, β=1/kT (with *k* being the Boltzmann constant and *T* the temperature), *G*(*x*) is the free energy at state *x*, and *Z* is the partition function.

The free energy at each point in the multi-dimensional space is computed using the probabilities derived from the histograms:

(2) $$G\left(x\right)=-kTln\left(P(x)\right)+C$$

where *C* is a constant that can be adjusted based on reference states.

#### 4.2.2. Calculation of the Binding Free Energy for Protein-DNA Interactions

The MM-PBSA (Molecular Mechanics Poisson Boltzmann Surface Area) or MM-GBSA (Molecular Mechanics Generalized Born Surface Area) methods in AMBER20 are widely used approaches for estimating the binding free energy of biomolecular complexes, such as protein-ligand interactions [44]. These methods combine molecular mechanics with continuum solvation models to provide a comprehensive evaluation of the free energy changes associated with binding. In this work, the binding free energy was calculated with the MM-GBSA method by the parallel version MMPBSA.py in AMBER20 [45]. The binding free energy (Δ*G_bind_*) can be expressed as the difference in free energy between the complexed and uncomplexed states [46]:

(3) $$∆G_{bind}=G_{complex}-\left(G_{receptor}-G_{ligand}\right)\;$$

where *G_complex_* is the free energy of the receptor-ligand complex, *G_receptor_* is the free energy of the receptor in isolation, and *G_ligand_* is the free energy of the ligand in isolation.

### 4.3. Dynamic Cross-Correlation Analysis

#### 4.3.1. Covariance Calculation

The dynamic cross-correlation analyses were calculated with GROMACS-2022 tools and Python-3.9 scripts [43]. First, we utilized the “gmx covar” tool within the GROMACS-2022 software to construct the dynamic cross-correlation matrices (DCCM) for each system. Subsequently, we employed Python-3.9 scripts to calculate the dynamic cross-correlation coefficients (DCCs) between the CA atoms of each residue. The motion pattern correlation between residues can be determined by calculating the covariance between pairwise residues. The formulas are as follows:

(4) $$c\left(i,j\right)=\langle ∆R_{i}\cdot ∆R_{j}\rangle \;$$

where

(5) $$∆R_{i}=R_{i}-\langle R_{i}\rangle \;$$

Here, *i* and *j* represent the residue indices of the protein amino acids, and *c*(*i*, *j*) is the covariance between residue *i* and residue *j*. The angle brackets denote an ensemble average, which means the average over a series of models, including multiple frames from a simulation trajectory or multiple structures obtained from NMR, etc. Δ*R_i_* represents the positional deviation based on the ensemble average position.

#### 4.3.2. Correlation Coefficient Calculation

The dynamic correlation coefficients are the covariance standardized after removing the influence of the magnitude of changes in the two variables, reflecting the pure correlation between the two variables. These coefficients are used to construct the DCCM by converting the covariance between residue pairs into correlation indices and organizing them into a matrix according to residue numbering. The DCCs (*C*(*i*,*j*)) between two residues could be calculated using the following equation [47]:

(6) $$C\left(i,j\right)=\frac{c\left(i,j\right)}{{\left[c\left(i,i\right)\cdot c\left(j,j\right)\right]}^{1/2}}$$

### 4.4. Analyses of Contact Maps

The residue contact map was calculated based on distances between all heavy atoms in both the protein and DNA, with a cutoff distance of 4.5 Å set according to references [8,48]. In the complex structure, any two heavy atoms that are less than 4.5 Å apart were defined as a contact between the corresponding residues. The residue contact probability was then calculated as the ratio of frames containing the contacts to the total number of trajectory frames. For Root Mean Square Fluctuation (RMSF) calculations, we first aligned all structures to the initial reference structure by superimposing the DBD (residues 92–289). The RMSF values were then computed for the heavy atoms of each residue.

## 5. Conclusions

In summary, this work elucidates a hierarchical model of p53 regulation wherein DNA binding initiates allostery and CTD interaction amplifies and refines it. Future studies exploring the role of the N-terminal domain and the effects of post-translational modifications on this network will further illuminate the full spectrum of p53 regulation. Ultimately, understanding the dynamic interplay among disordered regions and structured domains in p53 opens pathways for allosteric drug discovery and offers a paradigm for studying multi-domain proteins with intrinsically disordered regions.

## Figures and Tables

**Table 1:** The four molecular dynamics simulation systems.

Systems	States	Residues	Time
p53_DBD_	Protein	92–290	1 μs × 2
p53_DBD-DNA_	Protein-DNA	92–290	1 μs × 2
p53_ALL_	Protein	1–393	1 μs × 2
p53_ALL-DNA_	Protein-DNA	1–393	1 μs × 2

## Data Availability

The original contributions presented in this study are included in the article/Supplementary Material. Further inquiries can be directed to the corresponding authors.

## Supplementary Materials

The following supporting information can be downloaded at: https://www.mdpi.com/article/10.3390/ph19010124/s1, Figure S1: Domain structure of p53 and the internal interaction of p53 domains; Figure S2: Changes in the interaction interfaces of p53DBD and p53ALL upon DNA binding; Figure S3: Dynamic changes of internal interactions within the p53 DBD following DNA binding; Figure S4: The free energy landscape of p53DBD upon DNA binding; Figure S5: The free energy landscape change of p53 S6-S7 loop and L1 upon DNA binding; Figure S6: Conformational network changes of p53DBD and p53ALL before and after DNA binding; Figure S7: The correlation difference for the p53DBD upon DNA binding; Figure S8: The correlation difference for the p53ALL upon DNA binding; Figure S9: The free energy landscape of p53 p53ALL upon DNA binding; Figure S10: Changes in the secondary structure of the p53 N-terminal and C-terminal domains following DNA binding; Figure S11: Validation of the robustness of different p53 prediction models; Table S1: The binding free energy of p53 DBD and DNA; Table S2: The binding free energy of p53 and DNA; Table S3: The binding free energy of p53_R379A and DNA; Table S4: The binding free energy of p53_F212A-R280A and DNA.

## Abbreviations

IDRs	Intrinsically disordered regions
CTD	C-terminal domain
DBD	DNA-binding domain
RMSD	Root-mean-square deviation
RMSF	Root-mean-square fluctuation
MM-GBSA	Molecular mechanics generalized Born surface area
DCCs	Dynamic cross-correlation coefficients
FELs	Free energy landscapes
