# Supplementary material for: The Allosteric Regulation of the DNA-Binding Domain of p53 by the Intrinsically Disordered C-Terminal Domain
Source: Pharmaceuticals (Basel). 2026 Jan 10;19(1):124. doi: 10.3390/ph19010124 (PMC12845269; doi:10.3390/ph19010124)
Supplement: Supplementary file 1 [file pharmaceuticals-19-00124-s001.zip › pharmaceuticals-4033429-supplementary.pdf]

Supplemental Material

The allosteric regulation of the DNA-binding domain of p53 by the intrinsically disordered C-terminal domain

Shangbo Ning [1,†,*], Chengwei Zeng [2,†], Huiwen Wang [3], Junfeng Zhang [1], Yun Xue [1], and Yunjie Zhao [2,*]

[1] School of Medical Technology and Engineering, Henan University of Science and Technology, Luoyang, 471023, China

[2] Institute of Biophysics and Department of Physics, Central China Normal University, Wuhan, 430079, China

[3] School of Physics and Engineering, Henan University of Science and Technology, Luoyang, 471023, China

* Correspondence: yjzhaowh@mail.ccnu.edu.cn; sning@haust.edu.cn.

† These authors contributed equally to this work.

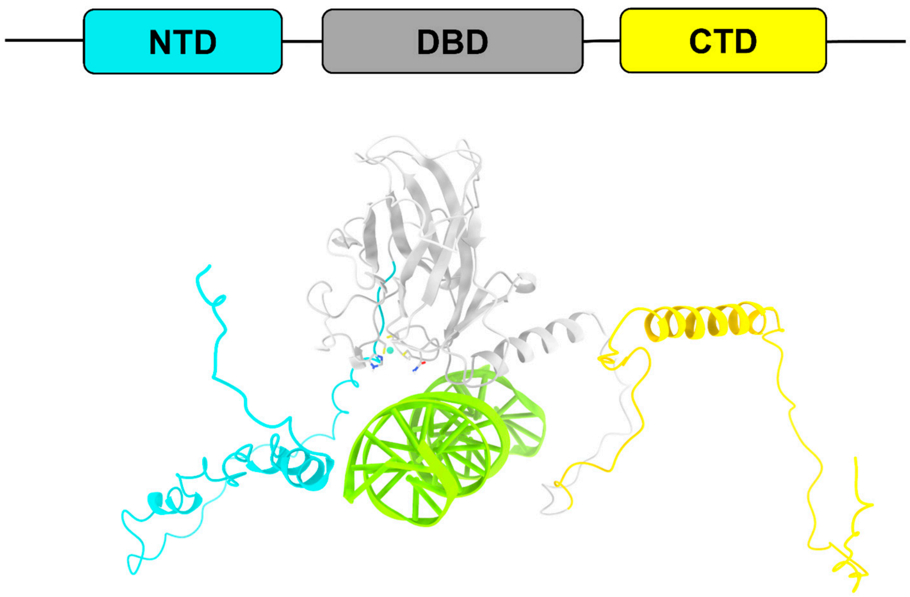

Figure S1. Domain structure of p53 and the internal interaction of p53 domains.

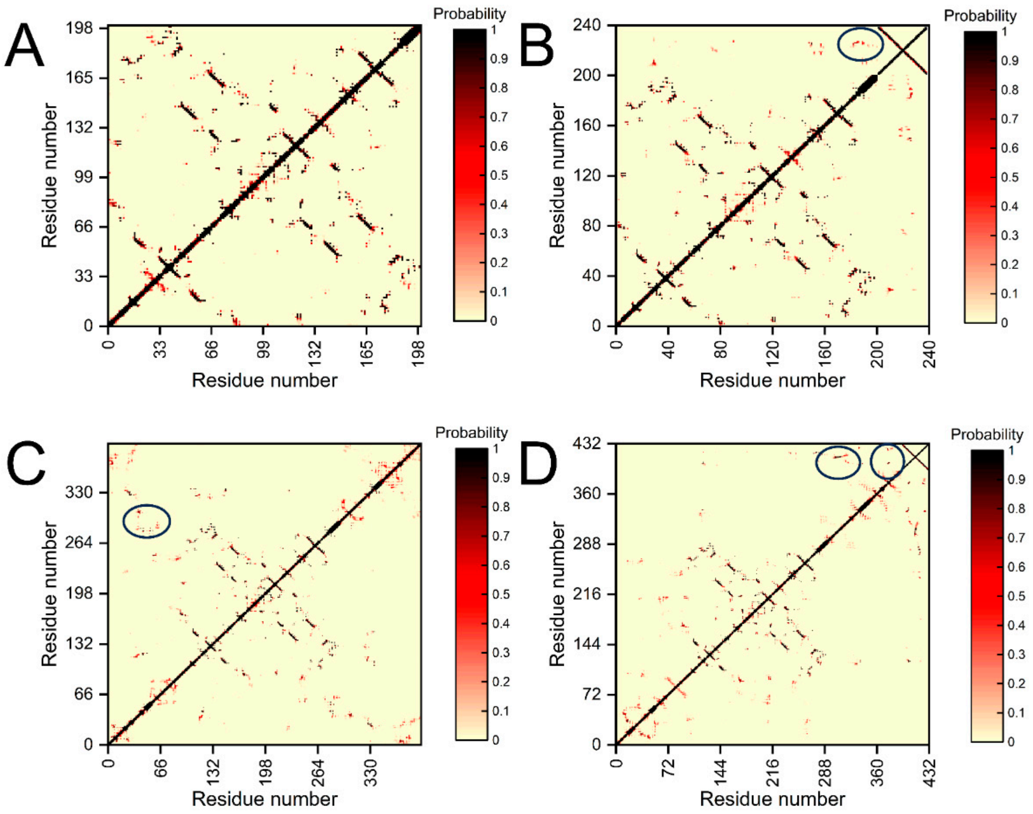

Figure S2. Changes in the interaction interfaces of p53DBD and p53ALL upon DNA binding. (A) Residue contact map of p53DBD; (B) Residue contact map of p53DBD-DNA; (C) Residue contact map of p53ALL; (D) Residue contact map of p53ALL-DNA.

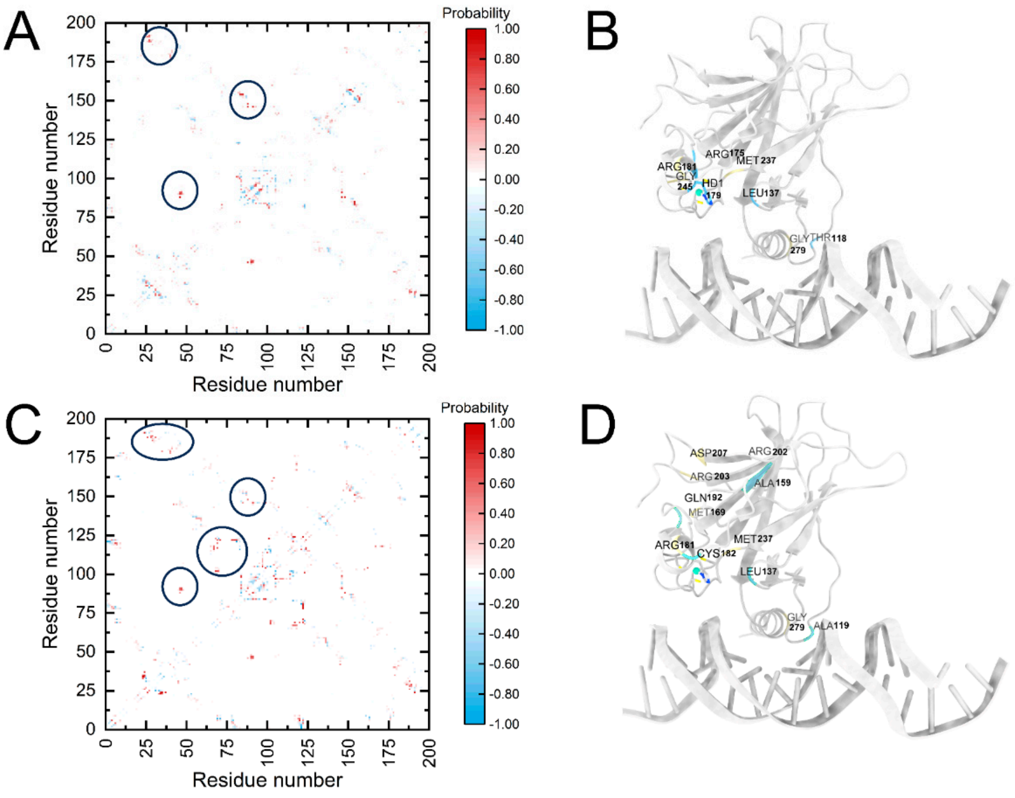

Figure S3. Dynamic changes of internal interactions within the p53 DBD following DNA binding. (A) Residue contact map of the p53DBD-DNA from simulations. (B) The sites of the increased interactions are shown in the p53DBD-DNA structure; (C) Residue contact map of the DBD domain in simulations of the p53ALL-DNA; (D) In simulations of the p53ALL-DNA, the regions with enhanced interactions after DNA binding are more obvious compared to those observed in p53DBD-DNA simulations.

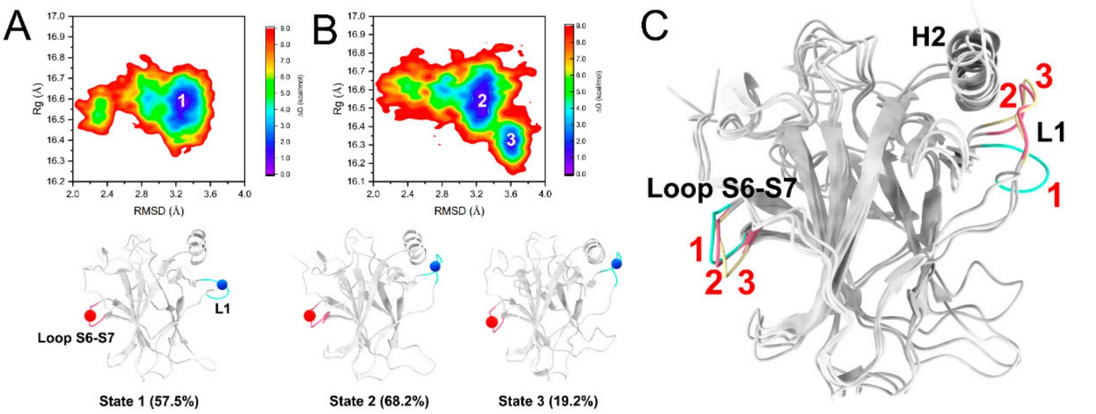

Figure S4. The free energy landscape of p53DBD upon DNA binding. (A) The 2D free energy landscape of p53DBD is displayed using two CVs, with the X-axis representing the RMSD changes of the DBD relative to the initial structure and the Y-axis representing the gyration radius of the DBD. The free energy surface describes the conformational changes and major conformational states of the DBD region during the simulation. (B) RMSD and Rg describe the free energy landscape of the DBD conformation of p53DBD-DNA. Upon binding to DNA, the conformation of the DBD shows two energy minima, and the population distribution also changes. (C) The overlap of the representative conformations of the three states for p53DBD upon DNA binding. The three conformational states of the DBD mainly differ in the conformational changes of the S6-S7 loop and the L1 loop.

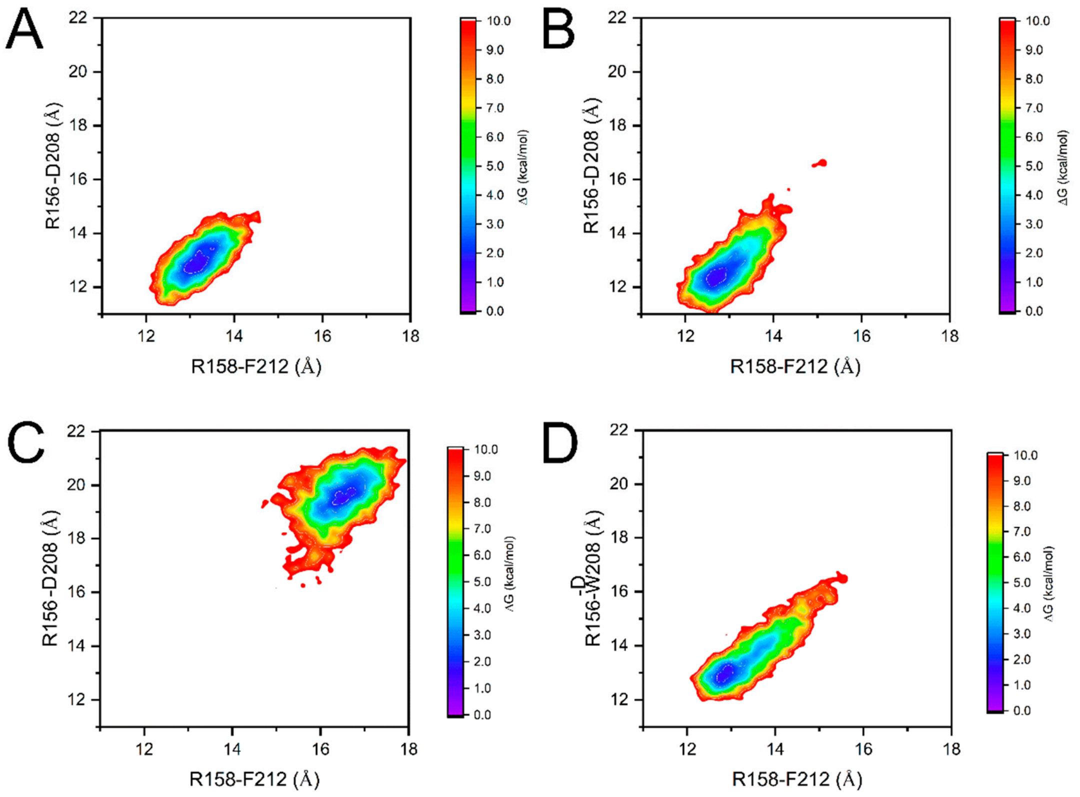

Figure S5. The free energy landscape change of p53 S6-S7 loop and L1 upon DNA binding. (A) The 2D free energy landscape of the p53DBD DBD conformation is displayed using two collective variables, with the X-axis representing the distance between the CA atoms of R158 and F212, and the Y-axis representing the distance between the CA atoms of R156 and D208. The free energy surface describes the conformational changes of the S6-S7 loop within the DBD. (B) The free energy landscape of the DBD conformation of p53DBD bound to DNA is characterized by the CA atom distances of R158-F212 and R156-D208. (C) The free energy landscape of the DBD conformation of p53ALL describes the conformational changes of the S6-S7 loop within the DBD. (D) The CA atom distances of R158-F212 and R156-D208 characterize the free energy landscape of the DBD conformation of p53ALL-DNA.

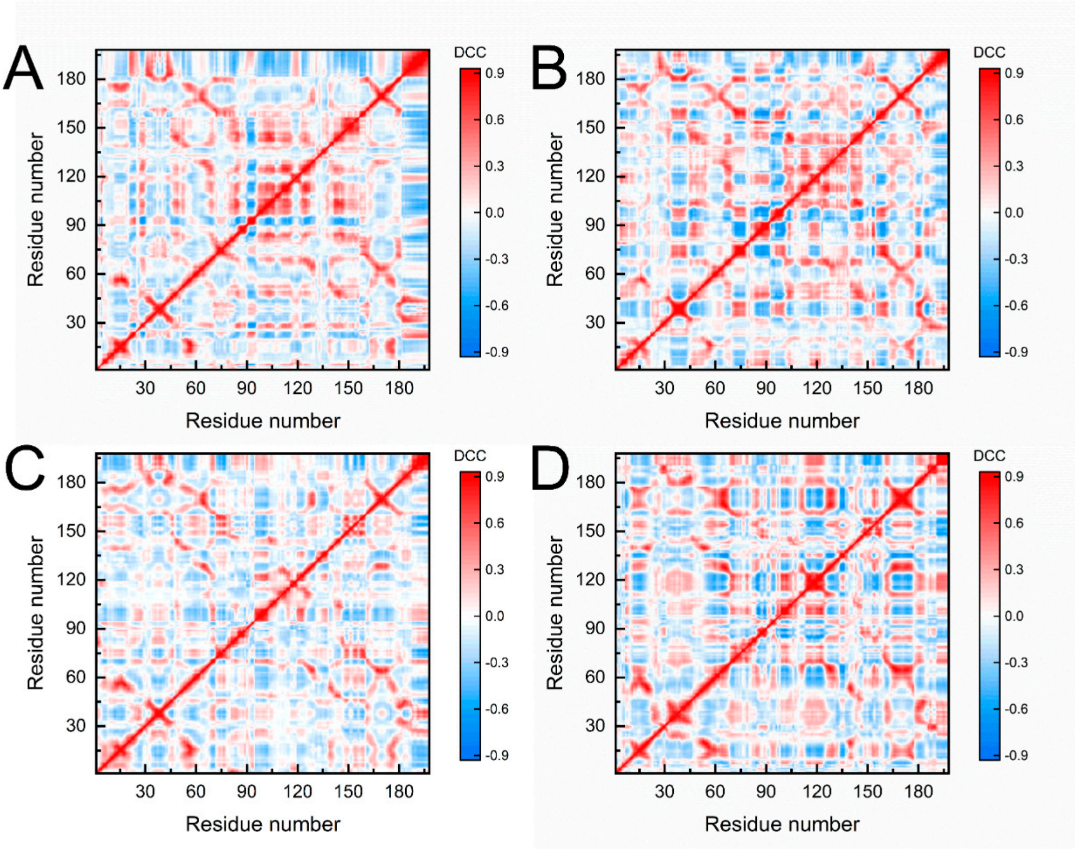

Figure S6. Conformational network changes of p53DBD and p53ALL before and after DNA binding. (A) Dynamic correlation coefficients of the DBD region in the p53DBD system; (B) Dynamic correlation coefficients of the DBD region in the p53DBD-DNA system; (C) Dynamic correlation coefficients of the DBD region in the p53ALL system; (D) Dynamic correlation coefficients of the DBD region in the p53ALL-DNA system.

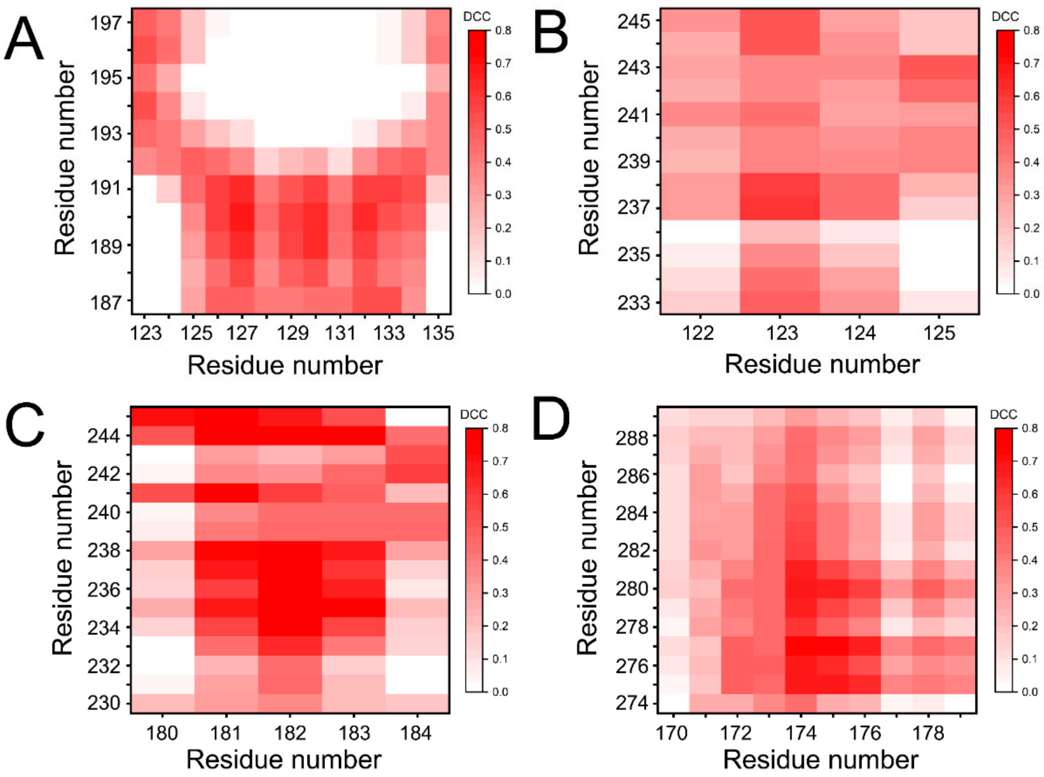

Figure S7. The correlation difference for the p53DBD upon DNA binding. (A), (B), (C), and (D) correspond to four regions with significant changes in the dynamic network. The red grids represent the increased correlations on the DBD upon DNA binding.

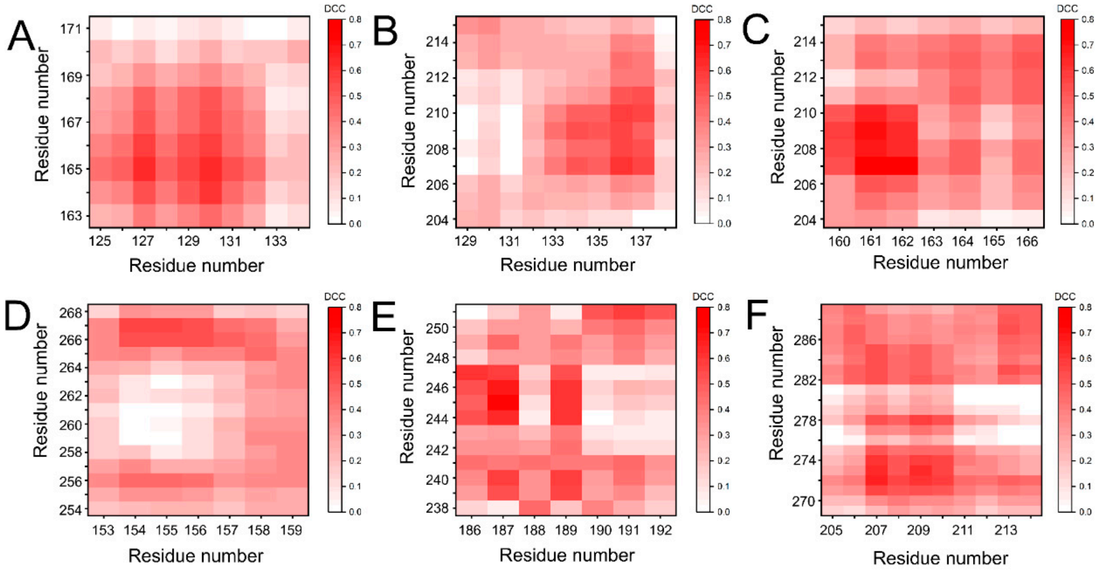

Figure S8. The correlation difference for the p53ALL upon DNA binding. (A), (B), (C), (D), (E), and (F) correspond to six regions with significant changes in the dynamic network. The red grids represent the increased correlations on the DBD upon DNA binding.

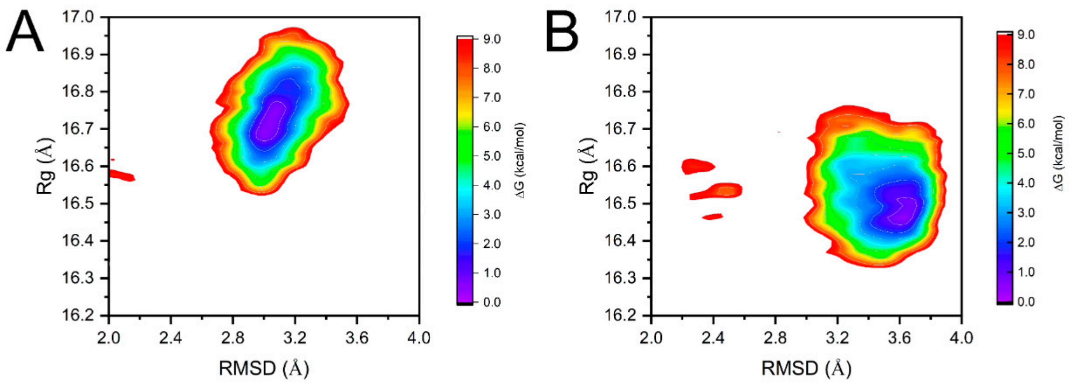

Figure S9. The free energy landscape of p53 $p53_{ALL}$ upon DNA binding. (A) The 2D free energy landscape of the DBD of the $p53_{ALL}$ is displayed using two collective variables, with the X-axis representing the RMSD changes of the DBD relative to the initial structure and the Y-axis representing the gyration radius of the DBD. The free energy surface describes the conformational changes and major conformational states of the DBD region during the simulation. (B) RMSD and Rg describe the free energy landscape of the DBD conformation of p53ALL-DNA.

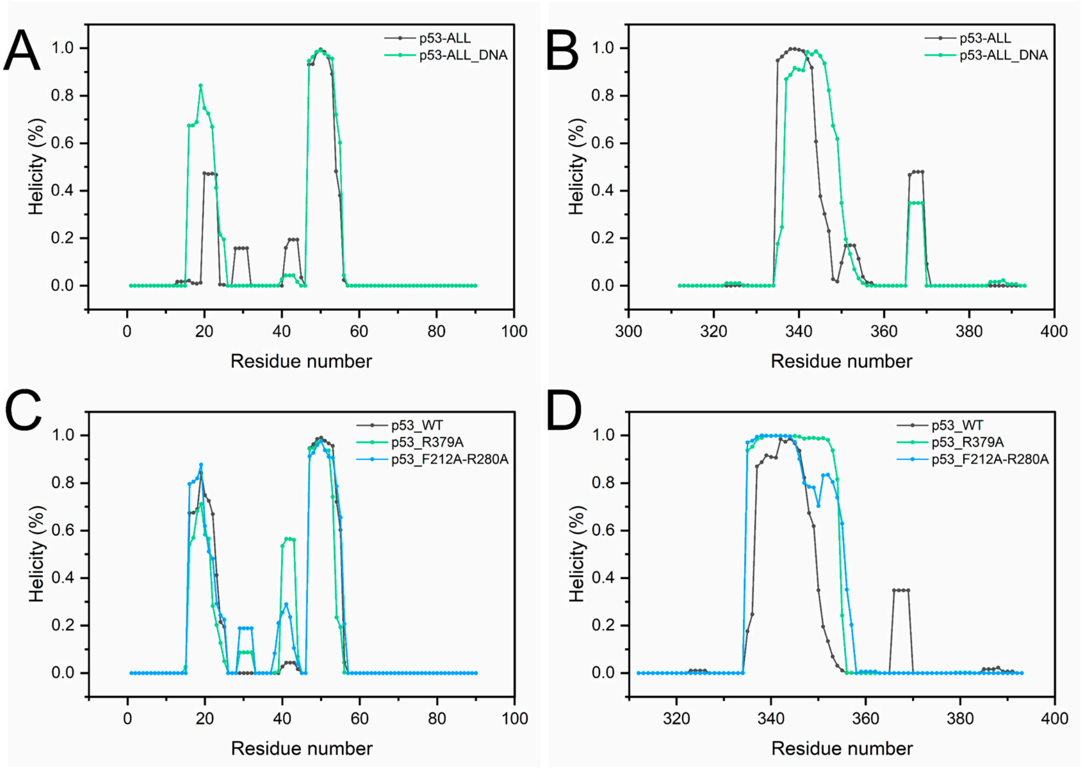

Figure S10. Changes in the secondary structure of the p53 N-terminal and C-terminal domains following DNA binding. (A) Helicity of the NTD in full-length wild-type p53 upon DNA binding. (B) Helicity of the CTD in full-length wild-type p53 upon DNA binding. (C) DNA binding-induced changes in NTD helicity for the p53 mutants R379A and F212A/R280A. (D) DNA binding-induced changes in CTD helicity for the p53 mutants R379A and F212A/R280A.

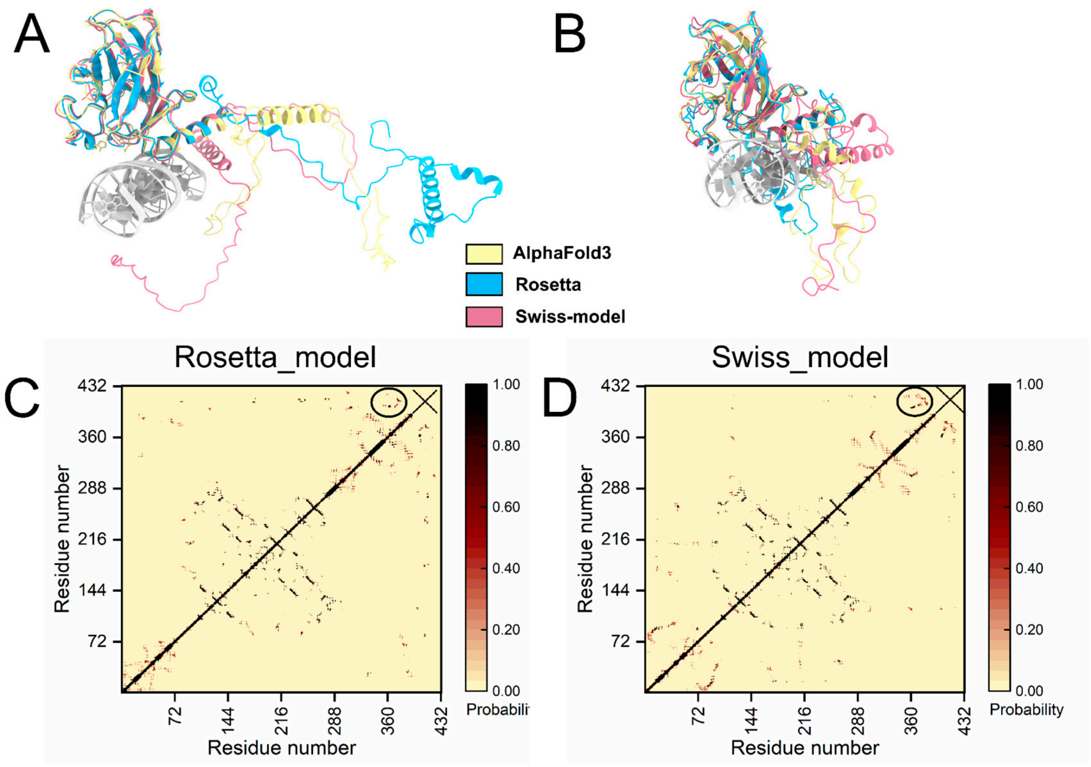

Figure S11. Validation of the robustness of different p53 prediction models. (A) Initial structures of the three prediction models; (B) Final structures of the molecular dynamics models for the three prediction models in the MD simulation; (C) Residue contact map of the Rosetta structure in the MD simulations; (D) Residue contact map of the Swiss-model structure. Molecular dynamics simulations of both predicted models reveal binding interactions between the CTD and DNA.

Table S1. The binding free energy of p53 DBD and DNA (kcal mol^{-1})

Energy component	Average	Std. Dev.	Std. Err. of Mean
E_{VDW}	-49.98	5.98	0.06
E_{ele}	-2634.57	181.58	1.82
E_{GB}	2651.17	175.89	1.76
E_{Surf}	-8.17	0.74	0.01
ΔG_{gas}	-2684.56	182.54	1.83
ΔG_{Solv}	2643.00	175.61	1.76
ΔG	-41.56	11.03	0.11

Table S2. The binding free energy of p53 and DNA (kcal mol^{-1})

Energy component	Average	Std. Dev.	Std. Err. of Mean
E_{VDW}	-148.88	28.92	0.29
E_{ele}	-4276.92	734.38	7.34
E_{GB}	4306.24	730.81	7.31
E_{Surf}	-21.49	3.60	0.04
ΔG_{gas}	-4425.80	744.71	7.45
ΔG_{Solv}	4284.74	729.39	7.29
ΔG	-141.06	34.48	0.34

Table S3. The binding free energy of p53_R379A and DNA (kcal mol^{-1})

Energy component	Average	Std. Dev.	Std. Err. of Mean
E_{VDW}	-104.97	7.76	0.10
E_{ele}	-3416.83	243.61	3.44
E_{GB}	3427.99	242.713	3.43
E_{Surf}	-14.77	0.82	0.01
ΔG_{gas}	-3521.80	244.62	3.45
ΔG_{Solv}	3413.22	242.67	3.43
ΔG	-108.57	14.18	0.20

Table S4. The binding free energy of p53_F212A-R280A and DNA (kcal mol^{-1})

Energy component	Average	Std. Dev.	Std. Err. of Mean
E_{VDW}	-132.30	10.64	0.15
E_{ele}	-3575.69	190.04	2.68
E_{GB}	3597.78	188.61	2.66
E_{Surf}	-18.78	1.26	0.01
ΔG_{gas}	-3707.99	192.88	2.72
ΔG_{Solv}	3579.00	188.09	2.66
ΔG	-128.99	14.70	0.20
